# Comparison of twelve‐layer concentric object (TLCO) and Line segmentations methods to determine the renal corticomedullary sodium gradient (CMSG) with a 3 T MRI

**DOI:** 10.1002/mrm.30525

**Published:** 2025-04-28

**Authors:** Patrik Jan Gallinnis, Rika Möller, Alexandra Ljimani, Yusuf Cabuk, Marie Scheuer, Charlotte Böttger, Cecilia Liang, Armin M. Nagel, Eric Bechler, Hans‐Jörg Wittsack, Anja Müller‐Lutz, Benedikt Kamp

**Affiliations:** ^1^ Department of Diagnostic and Interventional Radiology, Medical Faculty and University Hospital Düsseldorf Heinrich‐Heine‐University Düsseldorf Düsseldorf Germany; ^2^ Diagnostic and Interventional Radiology, Department of Radiology University Hospital of Tuebingen Tübingen Germany; ^3^ Institute of Radiology, University Hospital Erlangen Friedrich‐Alexander‐Universität Erlangen‐Nürnberg (FAU) Erlangen Germany; ^4^ Davison of Medical Physics in Radiology German Cancer Research Center (DKFZ) Heidelberg Germany; ^5^ Core Facility for Magnetic Resonance Imaging, Medical Faculty and University Hospital Düsseldorf Heinrich‐Heine‐University Düsseldorf Düsseldorf Germany

**Keywords:** corticomedullary ^23^Na gradient, kidney sodium MRI, sodium MRI^23^Na MR imaging, twelve‐layer concentric objects (TLCO) method

## Abstract

**Purpose:**

^23^Na MRI is a functional imaging technique that facilitates measurements of the renal corticomedullary sodium gradient (CMSG). The CMSG can be determined by a region of interest (ROI) in the renal parenchyma along the corticomedullary axis (Line method) or by dividing the renal parenchyma into concentric layers, using the twelve‐layer concentric objects (TLCO) method. The aim of this study was to investigate the differences, strengths, and weaknesses in determining the CMSG using these methods.

**Methods:**

Ten healthy volunteers were examined on a 3 T MRI‐system. ^23^Na images were acquired using a double‐tuned ^23^Na/^1^H surface coil and a golden angle (GA) density‐adapted 3D radial (DA‐3D‐RAD) sequence. The CMSG was determined with the Line and TLCO methods. Intra‐ and inter‐reader analyses were performed by two radiologists.

**Results:**

The evaluated CMSG by the two methods does not differ statistically significantly. Compared to the Line method, the TLCO method provides improved results in terms of reliability, precision, reproducibility, and concordance in intra‐ and inter‐reader analyses. A CMSG of (6.7 ± 2.6) mM/mm was determined using the TLCO method in the segmentation process with the lowest standard deviation.

**Conclusion:**

The TLCO method shows superior performance in determining the CMSG compared to the Line method. Accordingly, the use of the TLCO method is recommended for future renal CMSG studies.

## 1 INTRODUCTION

In 2017, 1.2 million deaths were attributed to chronical kidney diseases (CKD), with projections indicating an anticipated increase in incidence because of demographic change and a rise in cardiovascular diseases and diabetes.^1^, ^2^ Early detection of CKD is of paramount importance for prevention and treatment, given that it is often asymptomatic in its early stages.^2^ CKD is often associated with kidney inflammation, changes in the microstructure, insufficient oxygen supply, and altered hemodynamics.^3^ These aspects can be analyzed using different morphological or functional MRI methods including T_1_/T_2_ mapping, BOLD imaging, QSM, DWI, DTI, and ^23^Na‐MRI.^4^, ^5^, ^6^, ^7^ ^23^Na‐MRI can be used to determine the corticomedullary sodium gradient (CMSG) and has been investigated as a potential indicator of kidney diseases such as tubular necrosis, stenosis, and atrophy.^8^, ^9^ Furthermore, ^23^Na plays an essential role in the regulation of the water and electrolyte balance of the body.^10^ ^23^Na‐MRI uses endogenous ^23^Na nuclei as a signal source. The physical properties of ^23^Na nuclei such as very short relaxation times, low abundance and 800 to 5500 times lower SNR compared to ^1^H‐MRI make it challenging to achieve sufficient image quality in ^23^Na‐MRI.^11^ Therefore, special acquisition techniques and hardware are required to capture ^23^Na images. Based on the signal intensity in the ^23^Na images, ^23^Na concentrations can be calculated.^12^, ^13^, ^14^ In addition to the ^23^Na concentration, other effects can influence the image intensity such as partial volume effects, differences in sodium relaxation times and the coil sensitivity.^15^ These influences can be minimized by suitable correction methods. Because of the difficulty in accurately assessing the sodium concentration, various studies have introduced the term apparent tissue sodium concentration (aTSC).^15^, ^16^, ^17^ Based on the aTSC map, the CMSG can be determined. The CMSG was previously analyzed using a linear region of interest (ROI) through the renal parenchyma along the corticomedullary axis (Line method).^13^, ^14^, ^18^, ^19^ In the field of renal BOLD imaging, the twelve‐layer concentric objects (TLCO) segmentation technique was applied.^20^, ^21^

We hypothesize that the TLCO method can be transferred to determine the CMSG based on aTSC maps (TLCO method). The aim of this study was to investigate differences, strengths, and weaknesses in determining the CMSG with these methods in an intra‐ and inter‐reader study. We hypothesized, that the CMSG does not depend on the chosen method or the radiologist.

## 2 METHODS

### 2.1 Study population

Written informed consent was obtained from all participants and the study was approved by the local ethics committee (Ethics Committee, Medical Faculty of the Heinrich‐Heine‐University Düsseldorf, study number 2021–1393). In this prospective study seven female and three male healthy volunteers were examined (mean age 24 ± 2 years).

### 2.2 Experimental setup and sequence protocol

All measurements were performed on a 3 T MRI System (Siemens Magnetom Prisma, Siemens Healthineers). To obtain morphological reference images, an 18‐channel body coil and a 32‐channel spine coil (Body 18 SlideConnect and Spine 32 DirectConnect, Siemens Healthineers) were used in combination. Morphological reference images were acquired using a half‐Fourier acquisition single‐shot turbo spin echo (HASTE) sequence with a resolution of 0.7 × 0.7 × 3.0 mm^3^. A double‐tuned ^1^H/^23^Na‐surface‐coil (RAPID Biomedical) was used to acquire ^23^Na images and additional ^1^H images for image registration. Four reference phantoms with 4% agarose content by weight (ROTI Garose, Carl ROTH) and ^23^Na concentrations of 50, 75, 100, and 125 mM were placed behind the coil for aTSC calculations. A sensitivity profile of the double‐tuned coil was determined using homogeneous water phantoms containing 154 mM ^23^Na, positioned behind and in front of the coil.^16^ The acquisition parameters included an isotropic resolution of 3.0 mm, a pulse duration of 0.50 ms, a flip angle of 90°, echo times of 0.3 ms, recovery time of 60.0 ms, a number of spokes of 50.000, and 12 averages. In accordance with the sensitivity profile, a pixel‐based sensitivity correction of reference phantoms and in vivo data was performed by dividing the signal intensity of acquired ^23^Na images by the normalized sensitivity profile. The SNR was calculated as the ratio of signal mean to noise standard deviation in an ROI behind the coil.

All images, obtained with the double tuned coil were measured with a golden angle (GA) density‐adapted 3D radial (DA‐3D‐RAD) sequence developed by Nagel et al.^22^ Images were acquired (respectively for [^1^H/^23^Na] with an isotropic resolution of [1.0/3.0] mm, a pulse duration of [0.50/0.25] ms, a flip angle of [5.0/70.0] degrees, echo times of [1.0/0.15] ms, and a number of spokes of [30 000/50 000]). For both sequences, the readout time was 5.0 ms and the repetition time was 10.0 ms. The participants were positioned head‐first in the supine position. After the acquisition of the morphological images, the coil setup was modified and the volunteers were repositioned such that the right kidney was aligned with the isocenter and situated at the center of the 1H/^23^Na‐surface‐coil.

### 2.3 Image reconstruction and registration

^1^H and ^23^Na images were reconstructed with a custom‐written MATLAB script (The MathWorks).^22^ Gibbs ringing was reduced using a radial Hanning filter. To adapt the ^23^Na images to the reference images, the ^23^Na images were resized to a matrix size of 180 × 180 × 180 using nearest neighbor interpolation. The interpolated images were used for CMSG determination. The ^23^Na images were registered with the sensitivity profile and spatial dependent sensitivity correction was performed. Pixels with a signal intensity lower than 10% of the measured maximum signal intensity were excluded. The image registration was performed in ITKsnap 3.8.0 by manually merging the ^1^H images to the acquired morphological HASTE images.^23^ The corresponding registration transformation was used to register the aTSC images.

### 2.4 aTSC determination

A linear regression of the phantom signal intensity as a function of the contained concentration was performed. An aTSC map was calculated based on the determined slope m in the linear regression and the corresponding signal intensity S(^23^Na). Because of relaxation effects, a correction factor c is necessary for aTSC determination.^15^ The aTSC map was obtained by

$$\text{aTSC}\;\left[x_{i},y_{i}\right]=\frac{c\cdot S\left(23\mathrm{Na}\right)\left[x_{i},y_{i}\right]}{m},$$

with the slope m in $\frac{1}{\mathrm{mM}}$. The correction factor c depended on the properties of the kidney (k) and the agarose (a). Assuming a bi‐exponential, $T_{2}^{*}$ decay c was calculated by $\frac{c^{\left(a\right)}}{c^{\left(k\right)}}$, where^17^, ^24^, ^25^

$$c^{\left(a/k\right)}=\left(1-e^{-\frac{\mathrm{TR}}{T_{1}^{\left(a/k\right)}}}\right)\cdot \left(\left(f\cdot e^{-\frac{\mathrm{TE}}{T_{2,s}^{*\left(a/k\right)}}}\right)+\left(1-f\right)\cdot e^{-\frac{\mathrm{TE}}{T_{2,l}^{*\left(a/k\right)}}}\right).$$

The bi‐exponential $T_{2}^{*}$ decay was subdivided into a short and long component ($T_{2,s}^{*}$ and $T_{2,l}^{*}$ respectively).^25^ f describes the ratio between both components. We used f = 0.6, $T_{1}^{\left(k\right)}$ = 34.0 ms, $T_{2,s}^{*\left(k\right)}/T_{2,l}^{*\left(k\right)}$ = (2.2/20.4) ms, $T_{1}^{\left(a\right)}$ = 38.5 ms, and $T_{2,s}^{*\left(a\right)}$/$T_{2,l}^{*\left(a\right)}$ = (6.0/13.0) ms for aTSC calculations.^16^, ^24^, ^26^, ^27^

### 2.5 Segmentation

All segmentations in the kidney were performed in the coronal layer posterior to the renal pelvis. The cortex and medullary pyramids were segmented by P.J.G. (medical physicist, 2 years experience) under supervision of A.L. (radiologist, 10 years experience) with the software ITKsnap 3.8.0.^23^ The TLCO and Line method for CMSG determination were carried out by Y.C., who is mentioned here as the first radiologist, and M.S. as the second radiologist. Both have 2 years of experience. For intra‐reader analysis the dataset was segmented twice by Y.C. The radiologist and segmentation are noted (e.g., as “1/2” for the first radiologist and the second segmentation).

### 2.6 Line method

The Line method was performed on the ^23^Na images with the medulla and cortex outlined. For each volunteer, the radiologist draws a line ROI through an arbitrary medulla. The aTSC within the drawn linear ROI was regridded using a nearest neighbor interpolation to a matrix with a width and depth of 3 pixels/9 mm and the corresponding drawn pixel length. Subsequently, the CMSG was determined by a linear regression of the averaged aTSC along the drawn line up to the maximum signal intensity.^12^

### 2.7 TLCO method

The TLCO algorithm was applied to the anatomical image by manually segmenting the renal parenchyma and transferring the automatic generated concentric layers to ^23^Na images. Afterward, the aTSC was averaged for each concentric ROI and plotted against the distance from the cortex. The CMSG was then calculated from the outer layers up to the layer with the maximum aTSC.

### 2.8 Statistical analysis

Statistical analysis was conducted in Python 3.10 using the scipy 1.11.1 library.^28^ After the data was tested for normal distribution using a Shapiro–Wilk test, significant differences were determined using a paired *t* test (P_pt_) between the averaged aTSCs in medulla and cortex for each volunteer. All statistical testing was conducted with a significance level of *p* ≤ 0.05. Bland–Altmann analysis were used to quantify the bias between Line and TLCO CMSG methods. A one‐way analysis of variance test was conducted to determine whether there were significant differences on CMSG depending on the radiologist and segmentation method.

Both methods were evaluated regarding agreement and reproducibility in an inter‐reader and intra‐reader analysis using Lin's concordance correlation coefficient (ρ). The agreement and reproducibility were classified based on McBride et al.^29^ as follows: for ρ < 0.90 as poor, for 0.90 < ρ < 0.95 as moderate, for 0.95 < ρ < 0.99 as substantial, and for 0.99 < ρ as almost perfect. The precision (as a linear correlation in the inter‐ and intra‐reader analysis) was quantified by the amount of the Pearson's correlation coefficient (r), with r < 0.1 none, 0.1 < r < 0.3 poor, 0.3 < r < 0.5 fair, 0.5 < r < 0.6 moderate, 0.6 < r < 0.8 moderate strong, and 0.8 < r < 0.9 as very strong and 0.9 < r as perfect.^30^, ^31^ Intra‐reader and inter‐reader reliabilities were both quantified by a two‐way mixed single measure correlation coefficient (ICC(3,1)) (r_i_).^32^ Based on the work of Koo and Li,^33^ the reliability was categorized as follows r_i_ < 0.5 poor, 0.5 < r_i_ < 0.75 moderate, 0.75 < r_i_ < 0.9 good, and 0.9 < r_i_ excellent. 95% confidence intervals (CI) were determined using Fisher z‐transformation.

## 3 RESULTS

**FIGURE 1:**
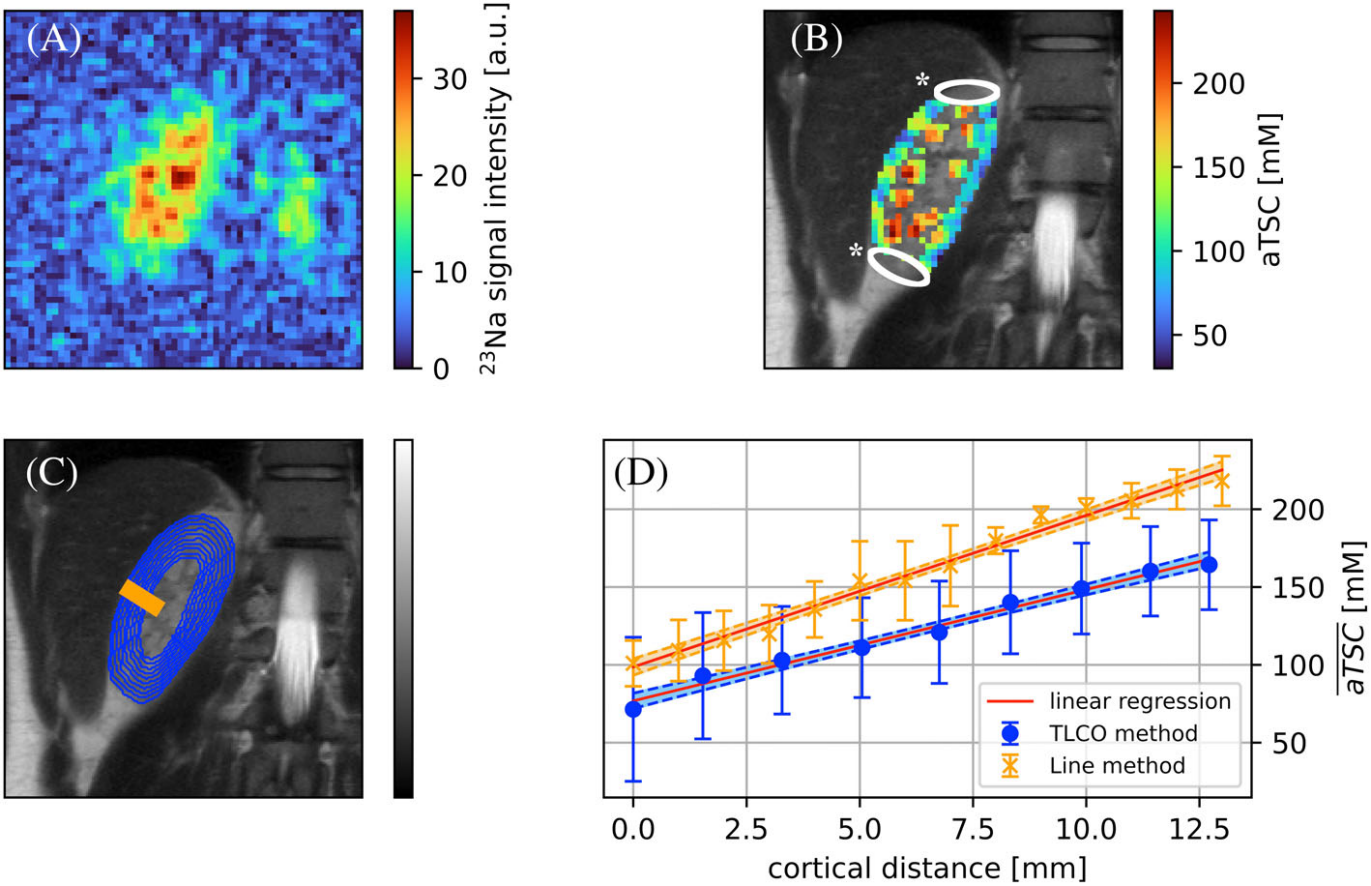
Representative illustration of the evaluation progress. Based on the acquired ^23^Na images (A), an apparent tissue sodium concentration (aTSC) map of a healthy volunteer (B) was calculated. An aTSC of (103.5 ± 31.7) mM was determined in the cortex and (166.1 ± 38.3) mM in the medulla. The white regions of interest (ROIs) indicated by * shows the area of pixels excluded by the sensitivity profile threshold of 10%. Based on the evaluated areas (C) defined by the Line (orange line) and twelve‐layer concentric object (TLCO) method (blue concentric circles) the corticomedullary sodium gradient (CMSG) was calculated using a linear regression model (D). The calculated CMSG were (9.7 ± 0.1) mM/mm and (7.2 ± 0.1) mM/mm, respectively, for Line and TLCO method.

In ^23^Na images, a SNR of 12.12 ± 2.58 in the medulla and 6.90 ± 1.35 in the cortex was determined (exemplary ^23^Na image in Figure 1A). The averaged aTSCs across all volunteers resulted in an aTSC of (137.2 ± 31.3) mM in the cortex and (202.3 ± 47.3) mM in the medulla (exemplary aTSC map in Figure 1B). A significant difference in the aTSC between medulla and cortex was found (P_pt_ <0.0001).

The application of the TLCO method permitted the evaluation of a larger cortical distance than that achievable with the Line method (Table 1). Furthermore, a stronger linear relationship was identified with the TLCO method than with the Line method. No significant differences were obtained in the determined CMSG, neither between the radiologists nor the segmentation method (P_pt_ >0.05).

**TABLE 1:** Summarized CMSG [mM/mm] results (mean ± SD) and evaluated distance along the renal parenchyma for each segmentation evaluation with the corresponding r^2^ of the linear regression of 10 healthy volunteers.

Method	Radiologist/segmentation	CMSG [mM/mm]	Distance [mm]	r^2^
TLCO	1/1	7.2 ± 3.1	15.1 ± 3.7	0.91 ± 0.12
	1/2	6.7 ± 2.6	14.9 ± 3.0	0.91 ± 0.09
	2/1	7.5 ± 3.2	14.4 ± 2.9	0.92 ± 0.09
Line	1/1	7.4 ± 2.3	11.6 ± 4.5	0.88 ± 0.11
	1/2	6.6 ± 3.7	13.5 ± 3.0	0.82 ± 0.22
	2/1	7.8 ± 3.4	13.3 ± 4.8	0.90 ± 0.10

Abbreviation: CMSG, corticomedullary sodium gradient.

**FIGURE 2:**
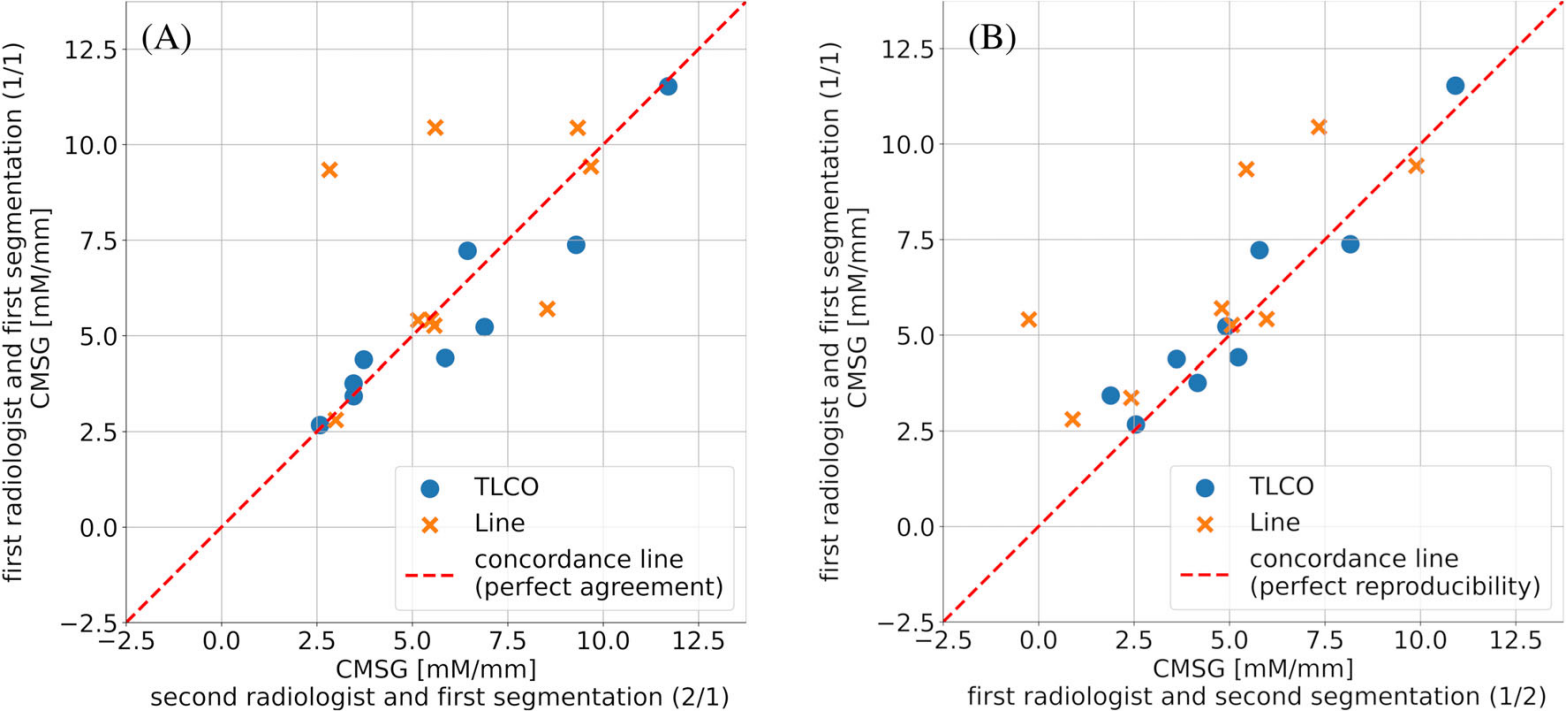
Concordance plots of inter‐reader (A) and intra‐reader (B) analysis for corticomedullary sodium gradient (CMSG) determined with the twelve‐layer concentric objects (TLCO) (blue dots) and line method evaluation (orange crosses).

The Bland–Altman analysis indicates a slight bias of the TLCO method, with a mean difference of 0.2 mM/mm compared to the Line method. The TLCO method provides moderate reproducibility for intra‐reader analysis (ρ = 0.92) and substantial agreement for inter‐reader analysis (ρ = 0.97), whereas the Line method provides poor reproducibility and agreement (ρ < 0.90) (Table 2 and Figure 2). The Pearson's correlation coefficient (r) indicates a very strong precision of the TLCO method for the inter‐ and intra‐reader analysis (r = 0.97 and r = 0.95, respectively). A poor precision was determined for the Line method in inter‐reader analysis (r = −0.13) whereas the precision was strong in the intra‐reader analysis (r = 0.78). The TLCO method shows for inter‐ and intra‐reader analysis an excellent reliability (r_i_ = 0.95). A poor reliability (r_i_ = 0.25) in both the intra‐ and inter‐reader analysis was obtained for the Line method. There were no significant differences (P_pt_ >0.05) between Line and TLCO methods for the intra‐ and inter‐reader analysis.

**TABLE 2:** Summarized results of the CMSG (mean ± SD) and statistical analysis of the inter‐ and intra‐reader analysis.

	Inter‐reader analysis		Intra‐reader analysis	
	TLCO method	Line method	TLCO method	Line method
CMSG [mM/mm]	7.3 ± 3.0	7.6 ± 2.8	6.9 ± 2.7	7.0 ± 3.0
ρ [CI]	0.97 [0.86–0.99]	−0.12 [−0.70 to 0.55]	0.92 [0.67–0.98]	0.67 [0.07–0.91]
r_i_ [CI]	0.95 [0.86–0.99]	0.25 [−0.12 to 0.68]	0.95 [0.86–0.99]	0.25 [−0.12 to 0.68]
r [CI]	0.97 [0.88–0.99]	−0.13 [−0.70 to 0.55]	0.95 [0.86–0.99]	0.78 [0.29–0.94]
P_pt_	0.27	0.79	0.16	0.32
CV	41%	36%	40%	42%

Abbreviations: CI, 95% confidence intervals; CMSG, corticomedullary sodium gradient; CV, coefficient of variation; Ppt paired *t* test *p*‐value; ρ, Lin's concordance correlation coefficient; (r), Pearson's correlation coefficient; (r_i_), two‐way mixed single measure correlation coefficient (ICC(3,1)); TLCO, 12 layer concentric objects.

## 4 DISCUSSION

The use of the TLCO algorithm enabled the successful determination of a linear aTSC concentration gradient from the cortex to the medulla in all subjects. A similar linear increase in aTSC was also reported in the work of Grist et al.^34^ The article did not provide the slope of the gradient, which precludes a comparison of the CMSG. Because the results of the two segmentation methods in our study do not differ significantly, the results can be compared with previously published CMSGs determined by the Line method and the application of the TLCO method is possible for future studies. However, it is recommended that the TLCO method be used in future CMSG evaluations, as the intra‐ and inter‐reader results demonstrate enhanced reliability, precision, reproducibility and concordance in comparison to the Line method.

It has been shown by Moon et al.^12^ that CMSG differs between transplanted and native kidneys. As demonstrated by Haneder et al.,^35^ the CMSG varies depending on the specific radiotherapeutic approach used. This suggests that the CMSG could potentially be used to evaluate not only the physiology but also the efficacy of different radiotherapeutic modalities in the future. However, to enable the clinical applicability of renal ^23^Na imaging, it is essential to use a robust and objective methodology for the assessment of CMSG. Our results indicate that the TLCO method is more appropriate for establishing CMSG as a clinical parameter than the Line method.

The TLCO segmentation of the first radiologist in the second segmentation shows the lowest standard deviation (see Table 1, [6.7 ± 2.6] mM/mm). This result is lower than the published results by Moon et al.,^12^ which found a CMSG of (10.5 ± 0.9) mM/mm in native kidneys and (8.9 ± 1.5) mM/mm in transplanted kidneys with normal function. Our CMSG results are increased compared to the results of Haneder et al.,^14^ who studied the CMSG under the influence of water loading using Line segmentation methods. They determined a CMSG of (2.56 ± 0.38) mM/mm for pre‐waterload and (3.38 ± 0.35) mM/mm for post‐water load. The methodology between these published results and ours differs in that the CMSG determined by Haneder et al.^14^ was only evaluated within the first 10 mm from the cortex, whereas we evaluated the CMSG up to the aTSC maximum, similar to the methodology of Moon et al.^12^ (Table 1). The aTSC published by Moon et al.^12^ (192.2 ± 9.6) mM are in good agreement with our aTSC results in the medulla ([202.3 ± 47.3] mM).

Our study has limitations that need to be mentioned when comparing our results with published studies. First, the aTSC maps depend on the individual hydration status of the test subjects.^14^, ^18^ In the present study, hydration monitoring was not performed to determine the aTSC maps used to calculate the CMSG. Nevertheless, it is anticipated that the performance of the two methods will not be influenced to a significant extent by differences in hydration. Second, the current MR acquisition time is long (˜45 min) because of coil change, volunteers positioning, shimming, and the current acquisition protocol, which makes additional relaxation time measurements for aTSC calculations and clinical application difficult to access. Third, we anticipate that partial volume artifacts will have an impact on the aTSC in our ^23^Na data acquired with a 3‐mm isotropic resolution of the ^23^Na images, given that they might influence the results. However, an improvement in resolution would be associated with a decline in signal accompanied by a loss of SNR, which is currently the limiting factor.

The statistical evaluation shows that the TLCO method should be preferred to the Line method in further CMSG studies, because of better reliability, precision, reproducibility, and concordance. The improvements of the TLCO method over the Line method have already been demonstrated in BOLD studies evaluating the corticomedullary gradient in renal oxygenation.^20^, ^36^ We acknowledge that integrating the ^23^Na protocol from this study into clinical routine is currently limited by time constraints. Our work highlights the advantages of the TLCO algorithm over the commonly used line algorithm. Advances in high‐field MRIs, shorter echo times, and AI‐driven developments are pushing ^23^Na imaging toward potential clinical applications.^15^ Objective measurement methods are essential for clinical implementation. We have demonstrated the objectivity of the TLCO algorithm compared to the established line algorithm. This study, therefore, contributes to establishing CMSG with ^23^Na as a potential clinical parameter.

## 5 CONCLUSION

The CMSG results using the TLCO method are comparable to the Line method, but offer better reliability, reproducibility, and precision in intra‐ and inter‐reader analyses. Therefore, we recommend using the TLCO method in future CMSG studies.
